# Cognitive Impairment Before Atrial Fibrillation–Related Ischemic Events: Neuroimaging and Prognostic Associations

**DOI:** 10.1161/JAHA.119.014537

**Published:** 2020-01-04

**Authors:** Gargi Banerjee, Edgar Chan, Gareth Ambler, Duncan Wilson, Lisa Cipolotti, Clare Shakeshaft, Hannah Cohen, Tarek Yousry, Rustam Al‐Shahi Salman, Gregory Y. H. Lip, Keith W. Muir, Martin M. Brown, Hans Rolf Jäger, David J. Werring, the CROMIS‐2 Collaborators, Louise Shaw, Kirsty Harkness, Jane Sword, Azlisham Mohd Nor, Pankaj Sharma, Deborah Kelly, Frances Harrington, Marc Randall, Matthew Smith, Karim Mahawish, Abduelbaset Elmarim, Bernard Esisi, Claire Cullen, Arumug Nallasivam, Christopher Price, Adrian Barry, Christine Roffe, John Coyle, Ahamad Hassan, Caroline Lovelock, Jonathan Birns, David Cohen, L. Sekaran, Adrian Parry‐Jones, Anthea Parry, David Hargroves, Harald Proschel, Prabel Datta, Khaled Darawil, Aravindakshan Manoj, Mathew Burn, Chris Patterson, Elio Giallombardo, Nigel Smyth, Syed Mansoor, Ijaz Anwar, Rachel Marsh, Sissi Ispoglou, Dinesh Chadha, Mathuri Prabhakaran, Sanjeevikumar Meenakishundaram, Janice O'Connell, Jon Scott, Vinodh Krishnamurthy, Prasanna Aghoram, Michael McCormick, Paul O'Mahony, Martin Cooper, Lillian Choy, Peter Wilkinson, Simon Leach, Sarah Caine, Ilse Burger, Gunaratam Gunathilagan, Paul Guyler, Hedley Emsley, Michelle Davis, Dulka Manawadu, Kath Pasco, Maam Mamun, Robert Luder, Mahmud Sajid, Ijaz Anwar, James Okwera, Julie Staals, Elizabeth Warburton, Kari Saastamoinen, Timothy England, Janet Putterill, Enrico Flossman, Michael Power, Krishna Dani, David Mangion, Appu Suman, John Corrigan, Enas Lawrence, Djamil Vahidassr

**Affiliations:** ^1^ Department of Brain Repair and Rehabilitation Stroke Research Centre UCL Queen Square Institute of Neurology and the National Hospital for Neurology and Neurosurgery London United Kingdom; ^2^ Department of Neuropsychology National Hospital for Neurology and Neurosurgery Queen Square London United Kingdom; ^3^ Department of Statistical Science University College London London United Kingdom; ^4^ New Zealand Brain Research Institute Christchurch New Zealand; ^5^ Haemostasis Research Unit Department of Haematology University College London London United Kingdom; ^6^ Lysholm Department of Neuroradiology and the Neuroradiological Academic Unit Department of Brain Repair and Rehabilitation UCL Queen Square Institute of Neurology Queen Square London United Kingdom; ^7^ Centre for Clinical Brain Sciences School of Clinical Sciences University of Edinburgh United Kingdom; ^8^ Liverpool Centre for Cardiovascular Science University of Liverpool and Liverpool Heart & Chest Hospital Liverpool United Kingdom; ^9^ Aalborg Thrombosis Research Unit Department of Clinical Medicine Aalborg University Aalborg Denmark; ^10^ Institute of Neuroscience & Psychology Queen Elizabeth University Hospital University of Glasgow United Kingdom

**Keywords:** atrial fibrillation, brain ischemia, cerebral small vessel disease, cognitive impairment, vascular dementia, Atrial Fibrillation, Cognitive Impairment, Ischemic Stroke, Transient Ischemic Attack (TIA)

## Abstract

**Background:**

It is likely that a proportion of poststroke cognitive impairment is sometimes attributable to unidentified prestroke decline; prestroke cognitive function is also clinically relevant because it is associated with poor functional outcomes, including death. We investigated the radiological and prognostic associations of preexisting cognitive impairment in patients with ischemic stroke or transient ischemic attack associated with atrial fibrillation.

**Methods and Results:**

We included 1102 patients from the prospective multicenter observational CROMIS‐2 (Clinical Relevance of Microbleeds in Stroke 2) atrial fibrillation study. Preexisting cognitive impairment was identified using the 16‐item Informant Questionnaire for Cognitive Decline in the Elderly. Functional outcome was measured using the modified Rankin scale. Preexisting cognitive impairment was common (n=271; 24.6%). The presence of lacunes (odds ratio [OR], 1.50; 95% CI, 1.03–1.05; *P*=0.034), increasing periventricular white matter hyperintensity grade (per grade increase, OR, 1.38; 95% CI, 1.17–1.63; *P*<0.0001), deep white matter hyperintensity grade (per grade increase, OR, 1.26; 95% CI, 1.05–1.51; *P*=0.011), and medial temporal atrophy grade (per grade increase, OR, 1.61; 95% CI, 1.34–1.95; *P*<0.0001) were independently associated with preexisting cognitive impairment. Preexisting cognitive impairment was associated with poorer functional outcome at 24 months (mRS >2; adjusted OR, 2.43; 95% CI, 1.42–4.20; *P*=0.001).

**Conclusions:**

Preexisting cognitive impairment in patients with atrial fibrillation–associated ischemic stroke or transient ischemic attack is common, and associated with imaging markers of cerebral small vessel disease and neurodegeneration, as well as with longer‐term functional outcome.

**Clinical Trial Registration:**

URL: http://www.clinicaltrials.gov. Unique identifier: NCT02513316.

**Clinical Perspective**

**What Is New?**

- We reviewed data from over 1000 patients presenting with ischemic stroke or transient ischemic attack associated with atrial fibrillation, recruited as part of the prospective, multicenter observational CROMIS‐2 (Clinical Relevance of Microbleeds in Stroke 2) atrial fibrillation study.
- We found that preexisting cognitive impairment is common in patients presenting with atrial fibrillation–associated ischemic stroke or transient ischemic attack, affecting nearly a quarter of patients (24.6%).
- Preexisting cognitive impairment was associated with magnetic resonance imaging markers of cerebral small vessel disease (lacunes, white matter hyperintensities) and neurodegeneration (medial temporal atrophy), and was also associated with poorer functional outcome at 24 months, independent of the acute ischemic event.

**What Are the Clinical Implications?**

- Preexisting cognitive impairment might be underrecognized in patients presenting with ischemic stroke or transient ischemic attack associated with atrial fibrillation.
- It is important to identify preexisting cognitive impairment in these patients, as it appears to have prognostic implications.
- We used an informant questionnaire (the Informant Questionnaire for Cognitive Decline in the Elderly) to identify preexisting cognitive impairment, which might be a useful and relevant tool in acute stroke; further data validation of this tool is needed.

## Introduction

Poststroke dementia is common, affecting up to 41.3% of patients in hospital populations.^1^ Atrial fibrillation (AF) is increasingly recognized as a key risk factor for dementia, both in association with and independent of clinically overt ischemic stroke.^2^ It is likely that a proportion of poststroke cognitive impairment is attributable to unidentified prestroke decline.^1^ The pooled prevalence of prestroke dementia is estimated to be 14.4% (in hospital‐based cohorts, on the basis of data from 3 studies); it is associated with both neurodegenerative and vascular factors^1^, ^3^ and with poor functional outcome.^4^, ^5^, ^6^, ^7^, ^8^ Most data on the clinical and radiological associations of prestroke cognitive impairment are from small, single‐center studies in heterogeneous stroke populations, which might not be generalizable to AF‐related stroke populations.^9^, ^10^, ^11^, ^12^, ^13^, ^14^, ^15^, ^16^, ^17^, ^18^, ^19^ Most imaging studies have focused on global and regional atrophy measures and white matter changes, with limited descriptions of other important structural markers of small vessel disease (such as magnetic resonance imaging [MRI]‐visible perivascular spaces and cerebral microbleeds).

We investigated the prevalence and associations of preexisting cognitive impairment in patients with ischemic stroke or transient ischemic attack (TIA) associated with AF. We hypothesized that patients with preexisting cognitive impairment would (1) have more evidence of small vessel disease and neurodegeneration that those without and (2) show associations with poorer functional outcome at 24 months.

## Methods

### Patient Selection

This is a predefined substudy nested within CROMIS‐2 AF, a multicenter prospective observational study of patients with AF‐related cardioembolic stroke or TIA, as described previously.^20^, ^21^ Briefly, this was a study of adults (aged ≥18 years) presenting with ischemic stroke or TIA with nonvalvular AF (confirmed by electrocardiography), who were eligible to start anticoagulation following their ischemic event.^20^, ^21^ Patients who could not have an MRI scan, had contraindications to anticoagulation, or had previously received therapeutic anticoagulation, were excluded.^20^, ^21^ The study was approved by the National Research Ethics Service (IRAS reference 10/H0716/61). Written informed consent was obtained in all cases.

### Informant Questionnaire for Cognitive Decline in the Elderly

Preexisting cognitive impairment was identified using the 16‐item Informant Questionnaire for Cognitive Decline in the Elderly (IQCODE). The informant (defined as the patient's caregiver, family member, or friend) was asked to compare the patient's cognitive and functional performance from 10 years before their stroke or TIA with their performance just before their stroke or TIA. The 16‐item IQCODE includes 16 questions, each of which can be scored between 1 and 5; the total is then divided by 16 to provide the final score (range, 1.0–5.0). Preexisting cognitive impairment was defined as an IQCODE score >3.3; this threshold was based on data from a systematic review evaluating the diagnostic accuracy of the IQCODE for detecting clinically diagnosed dementia (of any cause) in secondary care environments.^22^ Preexisting cognitive impairment was defined as an IQCODE score >3.3.^22^ All patients with a baseline IQCODE were included in this analysis.

### Imaging

Imaging was performed acutely after the index ischemic event and completed locally at each study center in accordance with a standardized protocol, which has been published previously.^20^ Imaging was performed acutely after the index ischemic event and completed locally at each study center in accordance with a standardized protocol including axial T2, T2* gradient echo sequence, diffusion‐weighted imaging, coronal T1, and fluid‐attenuated inversion recovery (FLAIR) images.^20^ Sequence parameters were specified for T2* gradient echo sequence^20^; the remaining sequences were obtained according to local protocols.

All structural markers of cerebral small vessel disease were rated in accordance with consensus criteria,^23^ each measure was rated by a single individual blinded to all clinical information. Where possible, the hemisphere contralateral to the acute stroke was preferentially counted.

Previous cortical infarcts were identified using T2 and FLAIR sequences and confirmed as nonacute through comparison with diffusion‐weighted images. Lacunes were identified and counted on T2 and FLAIR sequences using definitions from the Standards for Reporting and Imaging of Small Vessel Disease criteria.^23^ White matter hyperintensities (WMHs) in deep and periventricular distributions were rated on T2 and FLAIR sequences using the Fazekas scale.^24^ MRI‐visible perivascular spaces in the basal ganglia and centrum semiovale were rated on T2 and FLAIR sequences using a previously described validated visual rating scale.^25^ Medial temporal atrophy (MTA) was rated on T1 or FLAIR coronal images using the Scheltens visual scale.^26^ Global cortical atrophy (GCA) was rated with the Pasquier scale using axial T1, FLAIR, or inverted T2 images.^27^ Cortical superficial siderosis was identified on T2* gradient echo sequences and classified as either focal, involving ≤3 sulci, or disseminated, involving ≥4 sulci.^28^ Cerebral microbleeds were rated using T2* gradient echo sequences using the Microbleed Anatomical Rating Scale.^29^

### Functional Outcomes Following the Index Ischemic Event

Functional outcome at 24 months was quantified using the modified Rankin scale (mRS) using multiple ascertainment methods to maximize follow‐up; these included postal questionnaires sent to patients and their general practitioners, and death notifications from NHS Digital (previously the Health and Social Care Information Centre).^20^ The mRS was dichotomized, with a score of ≤2 indicating independence.^30^

### Statistical Analysis

We investigated for selection bias by comparing characteristics of those with and without a baseline IQCODE. We then compared baseline clinical, demographic, and imaging findings in patients with and without preexisting cognitive impairment. For all continuous variables, data were reviewed for normality, and if normally distributed, the independent t test was used. If variables were ordinal or not normally distributed, the nonparametric Mann–Whitney U test was used. Chi‐squared or Fisher's exact tests were used for categorical variables.

The results of univariable comparisons were used to identify variables for inclusion in multivariable logistic regression models; variables with *P*<0.20 were included in the adjusted analyses, except for situations where variables both described the same phenomenon (eg, clinical history of previous ischemic events and imaging evidence of a previous cortical infarct). The presence of ≥1 acute diffusion‐weighted image lesions was included as a variable in all adjusted analyses for outcome, to control for the index event (ie, stroke or TIA). We adjusted for National Institutes of Health Stroke Scale (a clinical measure of stroke severity) and the presence of acute diffusion‐weighted image changes as measures of the acute cerebral ischemic event; we did not additionally adjust for immediate postevent mRS for reasons of collinearity. We instead adjusted for preevent mRS to capture baseline function. Each model considered only a single neuroimaging marker at a time.

Post hoc analyses were performed after excluding those with a preexisting clinical diagnosis of dementia or cognitive impairment, previous ischemic events, or intracerebral hemorrhage at study entry. This was to establish whether any findings in the cohort as a whole were driven by patients with these diagnoses, which are associated with cognitive impairment.

Statistical analyses were performed (GB) using Stata (Version 15).

## Results

Of the total number of patients included in the CROMIS‐2 AF study (n=1490), we included 1102 patients for whom a baseline IQCODE was available; 388 patients were excluded as baseline IQCODE data were not available. The included patients were less likely to be current smokers (9.8% versus 16.9%; *P*<0.0001), more likely to have a formal diagnosis of dementia or cognitive impairment (2.8% versus 1.6%; *P*=0.166), less likely to have had a previous intracerebral hemorrhage (0.4% versus 1.1%; *P*=0.116), and more likely to be taking an antiplatelet drug at study entry (53.7% versus 48.7%; *P*=0.094). The included patients also had a slightly higher educational age (mean, 16.4 versus 16.9 years; *P*=0.027).

Baseline characteristics for our cohort are shown in Table 1. In our cohort (n=1102), the mean age was 76.0 years (SD, 10.1), and 471 patients (42.7%) were women.

**Table 1:** Baseline Demographic and Clinical Characteristics

	All	Preexisting Cognitive Impairment		*P* Value
		Absent	Present	
n (%)	1102	831 (75.4)	271 (24.6)	···
Age at event, y, mean (SD)	76.0 (10.1)	74.9 (10.1)	79.2 (9.4)	<0.00001
Sex, female, n (%)	471 (42.7)	338 (40.7)	133 (49.1)	0.015
Hypertension, n (%)	684 (62.8)	499 (60.6)	185 (70.0)	0.009
Hypercholesterolemia, n (%)	496 (45.6)	370 (45.1)	126 (47.2)	0.555
Diabetes mellitus, n (%)	186 (16.9)	130 (15.7)	56 (20.7)	0.057
Smoking at study entry, n (%)	106 (9.8)	86 (10.5)	20 (7.6)	0.168
Heart failure, n (%)	48 (4.4)	30 (3.6)	18 (6.6)	0.034
Known AF, n (%)	356 (32.6)	255 (31.0)	101 (37.7)	0.042
Previous ischemic event, n (%)	205 (19.1)	134 (16.5)	71 (27.1)	<0.0001
Previous intracerebral hemorrhage, n (%)	4 (0.4)	2 (0.2)	2 (0.8)	0.254
Educational age, y, mean (SD)	16.4 (3.5)	16.6 (3.8)	15.7 (2.4)	0.0003
Admission NIHSS, median (IQR)	5 (2–10)	5 (2–10)	4.5 (2–9)	0.9185
Antiplatelet use, n (%)	575 (53.7)	413 (51.0)	162 (62.1)	0.002

Percentage values were calculated using the total number of patients for whom data were available as the denominator. *P* values are from independent t tests (age at event, educational age), Mann–Whitney U test (NIHSS), Fisher's exact test (previous intracerebral haemorrhage), or chi‐squared tests (remainder). AF indicates atrial fibrillation; IQR, interquartile range; NIHSS, National Institutes of Health Stroke Scale.

### Preexisting Cognitive Impairment and Associations

In our cohort, the mean IQCODE score was 3.2 (SD, 0.6; score range, 1.0–5.0), and 271 (24.6%) patients had IQCODE‐defined preexisting cognitive impairment, of whom 23 (8.5%) had a known diagnosis of dementia or cognitive impairment at study entry. When comparing baseline clinical and demographic characteristics (Table 1), those with IQCODE‐defined preexisting cognitive impairment were older (79.2 years versus 75.4 years), more likely to be female (49.1% versus 40.7%), and have a diagnosis of hypertension (70.0% versus 60.6%), diabetes mellitus (20.7% versus 15.7%), heart failure (6.6% versus 3.6%), prior AF (37.7% versus 31.0%), and previous ischemic events (27.1% versus 16.5%). They were more likely to be taking an antiplatelet before their index event (62.1% versus 51.0%), and had a lower educational age (mean, 15.7 versus 16.6 years).

The neuroimaging features are presented in Table 2. Those with preexisting cognitive impairment were more likely to have previous cortical infarcts and lacunes. They had higher grades of periventricular WMHs, deep WMHs, MRI‐visible perivascular spaces in the basal ganglia, MTA, and global cortical atrophy and were more likely to have multiple cerebral microbleeds. In multivariable logistic regression analysis (Table 3), in which each imaging predictor was considered separately, the presence of lacunes (OR, 1.50, 95% CI, 1.03–1.05), increasing periventricular WMHs (per grade increase, OR, 1.38; 95% CI, 1.17–1.63), deep WMHs (per grade increase, OR, 1.26; 95% CI, 1.05–1.51) and MTA (per grade increase, OR, 1.61; 95% CI, 1.34–1.95) grade were independently associated with preexisting cognitive impairment.

**Table 2:** Comparison of Imaging Features Between Those With and Without Preexisting Cognitive Impairment

	All	Preexisting Cognitive Impairment		*P* Value
		Absent	Present	
n (%)	1102	831 (75.4)	271 (24.6)	···
Imaging evidence of previous cortical infarct, n (%)	207 (18.8)	142 (17.1)	65 (24.1)	0.011
Lacunes, presence, n (%)	188 (17.3)	130 (15.8)	58 (22.1)	0.020
pvWMH grade, n (%)				
0	645 (58.5)	527 (63.4)	118 (43.5)	<0.00001
1	206 (18.7)	149 (17.9)	57 (21.0)	
2	195 (17.7)	125 (15.0)	70 (25.8)	
3	56 (5.1)	30 (3.6)	26 (9.6)	
dWMH grade, n (%)				
0	472 (42.8)	385 (46.3)	87 (32.1)	<0.00001
1	431 (39.1)	315 (37.9)	116 (42.8)	
2	129 (11.7)	94 (11.3)	35 (12.9)	
3	70 (6.4)	37 (4.5)	33 (12.2)	
CSO‐PVS grade, n (%)				
0	58 (5.4)	44 (5.4)	14 (5.4)	0.5043
1	486 (45.2)	361 (44.3)	125 (48.1)	
2	324 (30.1)	255 (31.3)	69 (26.5)	
3	174 (16.2)	128 (15.7)	46 (17.7)	
4	33 (3.1)	27 (3.3)	6 (2.3)	
BG‐PVS grade, n (%)				
0	70 (6.4)	54 (6.6)	16 (6.0)	0.0033
1	782 (71.6)	607 (73.7)	175 (65.3)	
2	183 (16.8)	130 (15.8)	53 (19.8)	
3	52 (4.8)	30 (3.6)	22 (8.2)	
4	5 (0.5)	3 (0.4)	2 (0.8)	
MTA grade, n (%)				
0	222 (22.0)	192 (24.9)	30 (12.6)	<0.00001
1	470 (46.5)	373 (48.4)	97 (40.6)	
2	229 (22.7)	161 (20.9)	68 (28.5)	
3	66 (6.5)	38 (4.9)	28 (11.7)	
4	23 (2.3)	7 (0.9)	16 (6.7)	
GCA grade, n (%)				
0	355 (32.6)	282 (34.3)	73 (27.3)	0.0078
1	469 (43.1)	354 (43.1)	115 (43.1)	
2	246 (22.6)	174 (21.2)	72 (27.0)	
3	19 (1.7)	12 (1.5)	7 (2.6)	
cSS, presence, n (%)	3 (0.3)	1 (0.1)	2 (0.7)	0.151
CMB, presence, n (%)	230 (20.9)	165 (19.9)	65 (24.0)	0.146
Presence of >1 CMB, n (%)	111 (10.1)	71 (8.5)	40 (14.8)	0.003

Percentage values were calculated using the total number of patients for whom data were available as the denominator. *P* values are from Mann–Whitney U tests (pvWMH, dWMH, CSO‐PVS, BG‐PVS, MTA, and GCA grades), Fisher's exact test (cSS), or chi‐squared tests (remainder). BG‐PVS indicates magnetic resonance imaging–visible perivascular spaces in the basal ganglia; CMB, cerebral microbleed; CSO, magnetic resonance imaging–visible perivascular spaces in the centrum semiovale; cSS, cortical superficial siderosis; dWMH, deep white matter hyperintensities; GCA, global cortical atrophy; MTA, medial temporal atrophy; pvWMH, periventricular white matter hyperintensities.

**Table 3:** Multivariable Logistic Regression for Imaging Predictors of Preexisting Cognitive Impairment

	OR	95% CI	*P* Value
Imaging evidence of previous cortical infarct, presence^a^	1.23	0.84–1.78	0.288
Lacunes, presence^a^	1.50	1.03–1.05	0.034
pvWMH, per grade increase	1.38	1.17–1.63	<0.0001
dWMH, per grade increase	1.26	1.05–1.51	0.011
BG‐PVS, per grade increase	1.16	0.92–1.47	0.212
MTA, per grade increase	1.61	1.34–1.95	<0.0001
GCA, per grade increase	1.06	0.86 to1.31	0.588
cSS, presence	8.21	0.72–94.5	0.091
CMB, presence	1.10	0.76–1.58	0.620
Presence of >1 CMB	1.49	0.93–2.38	0.093

Each model considered only a single neuroimaging marker at a time. BG‐PVS indicates MRI‐visible perivascular spaces in the basal ganglia; CMB, cerebral microbleed; cSS, cortical superficial siderosis; dWMH, deep white matter hyperintensities; GCA, global cortical atrophy; MTA, medial temporal atrophy; OR, odds ratio; pvWMH, periventricular white matter hyperintensities.

a Adjusted for age at event, sex, hypertension, diabetes mellitus, smoking, heart failure, known atrial fibrillation, educational age, and anti‐platelet use. Remaining models were adjusted for age, sex, hypertension, diabetes mellitus, smoking, heart failure, clinical history of previous ischemic events, known atrial fibrillation, educational age, and antiplatelet use.

### Functional Outcome Data

Outcome data at 24 months were available for 922 patients (83.7%) of whom 480 (52.1%) were functionally dependent (mRS >2). Preexisting cognitive impairment was associated with functional dependence at 24 months (n=157, 72.0% versus n=323, 45.9%) in univariable (unadjusted OR, 3.03; 95% CI, 2.18–4.23; *P*<0.0001), and multivariable analyses (OR, 2.43; 95% CI, 1.42–4.20; *P*=0.001), adjusted for age at event, sex, hypertension, hypercholesterolemia, diabetes mellitus, smoking, heart failure, clinical history of previous ischemic events, educational age, admission National Institutes of Health Stroke Scale, antiplatelet use, preevent mRS, and the presence of an acute diffusion‐weighted image lesion at study entry.

### Subgroup Analyses

We then repeated these analyses after excluding patients with a known clinical history of dementia, cognitive impairment, previous ischemic events, or intracerebral hemorrhage at study entry, to review whether the associations observed in the whole cohort were being driven by patients with these diagnoses (Tables S1 through S4). The results of univariable and multivariable associations with preexisting IQCODE were consistent with our main findings, except that the association with lacunes and preexisting cognitive impairment did not reach statistical significance in adjusted analyses.

## Discussion

In our large, multicentre prospective cohort of patient with AF‐associated ischemic stroke and TIA, we found that nearly a quarter of patients (24.6%) met IQCODE criteria for preexisting cognitive impairment; this was associated with the presence of lacunes, periventricular and deep WMHs, and medial temporal atrophy, but not with other structural markers of small vessel disease (MRI‐visible perivascular spaces, cortical superficial siderosis, or cerebral microbleeds). We also found that IQCODE‐defined cognitive impairment was associated with poorer functional outcome at 24 months.

Our findings in an AF‐associated cohort are in keeping with previous studies that have shown that preexisting cognitive impairment is associated with both neurodegenerative and vascular factors.^9^, ^10^, ^11^, ^12^, ^13^, ^14^, ^15^, ^16^, ^17^, ^18^, ^31^ AF is increasingly recognized as a key risk factor for dementia, both in association with and independent of clinically overt ischemic stroke.^32^, ^33^, ^34^, ^35^, ^36^, ^37^ Multiple mechanisms for this association have been proposed, including silent brain infarcts from recurrent embolization, cerebral hypoperfusion, chronic inflammation, and endothelial dysfunction or progression of preexisting cerebrovascular or neurodegenerative processes.^38^, ^39^, ^40^, ^41^, ^42^, ^43^, ^44^, ^45^, ^46^, ^47^, ^48^, ^49^, ^50^, ^51^ We found rates of preexisting cognitive impairment that were higher than many unselected stroke populations, and our rates of impairment were also higher than those reported in other AF cohorts.^1^, ^9^, ^10^, ^11^, ^12^, ^13^, ^14^, ^15^, ^16^, ^17^, ^18^, ^31^ This might reflect the variability in methods used to diagnose preexisting cognitive impairment, including different IQCODE thresholds.

Our finding that MTA is a common and prevalent finding in patients before stroke provides further evidence that this neuroimaging feature is important in AF‐related ischemic stroke and TIA. AF has been shown to be associated with lower hippocampal volumes and poorer memory and learning performance in stroke‐free individuals, and patients with AF have greater atrophy of their entorhinal cortex and medial temporal lobes, compared with those without.^50^, ^52^ The relationship between AF and global atrophy measures is less clear; while one study found that AF was associated lower brain volumes globally, others did not identify an association, although this might reflect the younger age of these latter cohorts.^50^, ^51^, ^53^ An association with MTA but not global cortical atrophy is in keeping with our results and implicates Alzheimer disease pathology in the cognitive impairment associated with AF, a proposal for which there is supporting longitudinal and pathological data.^2^, ^54^, ^55^, ^56^, ^57^, ^58^ Proposed mechanisms by which AF might contribute to Alzheimer disease pathology include β‐ and γ‐secretase inhibition, perivascular amyloid clearance failures and tau phosphorylation, all of which might be induced by AF‐related cerebral hypoperfusion.^2^ However, MTA can also be a feature of vascular pathology, and it might be that this is the dominant pathology in AF.^59^, ^60^

We also found an association between WMH severity and preexisting cognition. Although WMHs in patients with AF might simply be attributable to age or a shared vascular risk factor profile,^61^ there is evidence to suggest that there is an independent association.^62^ While WMHs are associated with poorer cognitive performance,^63^ the data relating to cognitive impairment in AF and WMHs is conflicting, with some studies showing no association.^50^, ^51^ We did not find an independent statistically significant association with imaging evidence of previous cortical infarcts, which might provide further evidence that embolism to the brain (either clinically overt or “silent”) is not the only mechanism contributing to cognitive impairment in these patients. The lack of association between preexisting cognitive impairment and other structural small vessel disease markers (MRI‐visible perivascular spaces and cerebral microbleeds) is in keeping with data from other populations, which show inconsistent associations between cognitive impairment and these markers.^64^ The presence of both neurodegenerative and vascular pathologies support the argument that, in patients with ischemic stroke, preexisting dementia is a manifestation of “brain aging”^3^ rather than attributable to one single pathological process; this is in contrast with cognitive impairment before spontaneous intracerebral hemorrhage, where cerebral small vessel diseases (in particular, cerebral amyloid angiopathy) are important.^19^, ^65^ This suggests that any future therapeutic strategies for cognitive impairment will need to be implemented early and before stroke to be effective.

We show that cognitive impairment before an ischemic event is important, as it influences subsequent functional outcome, independent of the acute ischemic event. This is in keeping with previous work from other centers, which has shown associations between prestroke dementia and poor functional outcomes.^4^, ^5^, ^6^, ^7^, ^8^ Appropriately identifying preexisting cognitive impairment can be important for decision making with regard to postevent rehabilitation.^66^ Questions remain about how best to diagnose preexisting cognitive impairment. The IQCODE has been used extensively^67^; our data provide more evidence that the IQCODE might be a useful and relevant tool in acute stroke, as in our cohort it identifies patients at risk of subsequent cognitive impairment and poorer functional outcomes. While it might seem counterintuitive to not assess the patient directly, this is often useful in stroke where patients are unable to engage in formal testing, for example, because of aphasia or reduced consciousness. IQCODE‐based estimates of cognition might prove more accurate than the potential overestimation resulting from acute patient testing (which can be influenced by intercurrent illness) and potential underestimation from formal dementia diagnoses. The association of IQCODE‐defined cognitive impairment with recognized neurodegenerative and vascular neuroimaging markers suggests that this is indeed reflective of significant underlying pathology.

### Strengths and Limitations

The strengths of this study include its large size and multicenter design. We considered a wide range of structural markers associated with cerebral small vessel disease and neurodegeneration. However, there are also some limitations. Patients without IQCODE data (and therefore excluded from our analysis) were more likely to have a formal diagnosis of dementia or cognitive impairment, suggesting some selection bias in our cohort. While the study imaging protocol required standardized sequences, there was still variability in how these sequences were obtained, as well as the MRI machines used by each center, and this could influence our imaging rating. This is particularly the case for cerebral microbleeds, where detection may have been influenced by magnetic resonance field strength; unfortunately, we did not have data on this. The mean age of our cohort is 76.0 years, and so these findings may not be generalizable to patients who have stroke at significantly younger ages; many of the brain pathologies that coexist and contribute to cognitive impairment are age related. We are unable to provide further data on whether patients had paroxysmal or persistent AF; there is some evidence that the former might have particular relevance for cognition in this context.^2^, ^58^, ^68^ We do not have follow‐up data on cardiac function for this cohort, and we acknowledge that this might have influenced functional outcome. Additionally, the CROMIS‐2 AF study included only patients with recent cardioembolic ischemic stroke or TIA who were eligible for anticoagulation and able to undergo MRI scanning; this may not be representative of other patient groups. As we discussed above, MTA is not specific to Alzheimer disease; we do not have data on other markers of Alzheimer disease pathology (measures of Aβ and tau, eg, using positron emission tomography or cerebrospinal fluid) and therefore cannot comment further on the extent to which pathologies associated with Alzheimer disease contribute to our findings. We also acknowledge that the IQCODE threshold used might not be equivalent to dementia and that a range of thresholds have been used in the past; the IQCODE has not been validated for prestroke impairment against a formal diagnosis of dementia, and we were unable to comment on this in our study. Formal neuropsychological testing of multiple domains would provide a more comprehensive assessment of cognition. Despite this, we would argue that cognitive impairment at the level identified by the IQCODE is still of relevance given that it is able to predict future outcomes in our cohort, although further validation is needed before the IQCODE can be used for this purpose in clinical practice.

## Summary

In this comprehensive imaging description of the factors associated with preexisting cognitive impairment in cardioembolic stroke and TIA, we report that preexisting cognitive impairment is common and associated with imaging markers of cerebral small vessel disease and neurodegeneration, as well as poorer functional outcomes at 24 months. We also provide evidence that the IQCODE might be useful as an acute tool in ischemic stroke and TIA, as it appears to identify those likely to have worse clinical outcomes. Future work validating the IQCODE in this context (including establishing the optimal threshold for these patients), together with further investigation of the factors that contribute to brain resilience and whether this can be influenced after ischemic injury, is needed.

## Appendix

### The CROMIS‐2 Collaborators

Louise Shaw, MD; Kirsty Harkness, MD; Jane Sword, MD; Azlisham Mohd Nor, MD; Pankaj Sharma, PhD; Deborah Kelly, MD; Frances Harrington, MD; Marc Randall, MD; Matthew Smith, MD; Karim Mahawish, MD; Abduelbaset Elmarim, MD; Bernard Esisi, MD; Claire Cullen, MD; Arumug Nallasivam, MD; Christopher Price, MD; Adrian Barry, MD; Christine Roffe, MD; John Coyle, MD; Ahamad Hassan, MD; Caroline Lovelock, DPhil; Jonathan Birns, MD; David Cohen, MD; L. Sekaran, MD; Adrian Parry‐Jones, PhD; Anthea Parry, MD; David Hargroves, MD; Harald Proschel, MD; Prabel Datta, MD; Khaled Darawil, MD; Aravindakshan Manoj, MD; Mathew Burn, MD; Chris Patterson, MD; Elio Giallombardo, MD; Nigel Smyth, MD; Syed Mansoor, MD; Ijaz Anwar, MD; Rachel Marsh, MD; Sissi Ispoglou, MD; Dinesh Chadha, MD; Mathuri Prabhakaran, MD; Sanjeevikumar Meenakishundaram, MD; Janice O'Connell, MD; Jon Scott, MD; Vinodh Krishnamurthy, MD; Prasanna Aghoram, MD; Michael McCormick, MD; Paul O'Mahony, MD; Martin Cooper, MD; Lillian Choy, MD; Peter Wilkinson, MD; Simon Leach, MD; Sarah Caine, MD; Ilse Burger, MD; Gunaratam Gunathilagan, MD; Paul Guyler, MD; Hedley Emsley, MD; Michelle Davis, MD; Dulka Manawadu, MD; Kath Pasco, MD; Maam Mamun, MD; Robert Luder, MD; Mahmud Sajid, MD; Ijaz Anwar, MD; James Okwera, MD; Julie Staals, PhD; Elizabeth Warburton, MD; Kari Saastamoinen, MD; Timothy England, MD; Janet Putterill, MD; Enrico Flossman, MD; Michael Power, MD; Krishna Dani, MD; David Mangion, MD; Appu Suman, MD; John Corrigan, MD; Enas Lawrence, MD; and Djamil Vahidassr, MD.

## Sources of Funding

The CROMIS‐2 study is funded by the Stroke Association and British Heart Foundation. Dr Banerjee holds a National Institute for Health Research (NIHR) Academic Clinical Fellowship and received funding from the Rosetrees Trust. Dr Banerjee receives funding from the National Institute for Health Research University College London Hospitals Biomedical Research Centre. Dr Al‐Shahi Salman is funded by an Medical Research Council senior clinical fellowship. Dr Brown's chair in Stroke Medicine is supported by the Reta Lila Weston Trust for Medical Research. Dr Werring receives research support from the Stroke Association, the British Heart Foundation, and the Rosetrees Trust. This work was undertaken at University College London Hospitals NHS Foundation Trust and University College London, which receives a proportion of funding from the Department of Health's National Institute for Health Research Biomedical Research Centres funding scheme.

## Disclosures

Dr Cohen has received institutional research support from Bayer; honoraria for lectures and an Advisory Board from Bayer, diverted to a local charity; and travel/accommodation expenses for participation in scientific meetings covered by Bayer. Dr Lip acts as a consultant for Bayer/Janssen, Bristol‐Myers Squibb/Pfizer, Medtronic, Boehringer Ingelheim, Novartis, Verseon, and Daiichi‐Sankyo and as a speaker for Bayer, Bristol‐Myers Squibb/Pfizer, Medtronic, Boehringer Ingelheim, and Daiichi‐Sankyo; no fees are directly received personally. Dr Werring has received honoraria for lectures or chairing from Bayer and Portola, and for consultancy from Bayer, Alnylam and Portola. The remaining authors have no disclosures to report.

## Supporting information


**Table S1.** Baseline Demographic and Clinical Characteristics
**Table S2.** Comparison of Imaging Features Between Those and Without Preexisting Cognitive Impairment
**Table S3.** Multivariable Logistic Regression for Imaging Predictors of Preexisting Cognitive Impairment
**Table S4.** Logistic Regression Models Reviewing Associations Between IQCODE‐Defined Preexisting Cognitive Impairment and Functional Outcome at 24 Months

## Data Availability

Analyses for the CROMIS‐2 (Clinical Relevance of Microbleeds in Stroke 2) study are ongoing; once all of these analyses are completed, the CROMIS‐2 Steering Committee will consider applications from other researchers for access to anonymized source data.
