# Supplementary material for: Cognitive Impairment Before Atrial Fibrillation–Related Ischemic Events: Neuroimaging and Prognostic Associations
Source: J Am Heart Assoc. 2020 Jan 4;9(1):e014537. doi: 10.1161/JAHA.119.014537 (PMC6988157; doi:10.1161/JAHA.119.014537)
Supplement: Supplementary file 1 — Table S1. Baseline Demographic and Clinical Characteristics Table S2. Comparison of Imaging Features Between Those and Without Preexisting Cognitive Impairment Table S3. Multivariable Logistic Regression for Imaging Predictors of Preexisting Cognitive Impairment Table S4. Logistic Regression Models Reviewing Associations Between IQCODE‐Defined Preexisting Cognitive Impairment and Functional Outcome at 24 Months [file JAH3-9-e014537-s001.pdf]

SUPPLEMENTAL MATERIAL

Table S1. Baseline demographic and clinical characteristics.

	All	Pre-existing cognitive impairment		p value
		Absent	Present	
n (%)	872	689 (79.0)	183 (21.0)	-
Age at event, years , mean (SD)	75.1 (10.2)	74.2 (10.2)	78.5 (9.7)	<0.00001
Sex, female, n (%)	368 (42.2)	280 (40.6)	88 (40.1)	0.070
Hypertension, n (%)	519 (60.1)	400 (58.6)	119 (66.1)	0.066
Hypercholesterolaemia, n (%)	361 (41.9)	286 (42.0)	75 (41.7)	0.936
Diabetes mellitus, n (%)	137 (15.8)	102 (14.9)	35 (19.1)	0.158
Smoking at study entry, n (%)	91 (10.6)	75 (11.0)	16 (8.9)	0.406
Heart failure, n (%)	33 (3.8)	21 (3.1)	12 (6.6)	0.027
Known AF, n (%)	271 (31.4)	206 (30.2)	65 (35.9)	0.138
Educational age, years, mean (SD)	16.5 (3.7)	46.7 (3.2)	15.7 (2.4)	0.0031
Admission NIHSS, median (IQR)	5 (2 to 10)	5 (2 to 10)	5 (2 to 10)	0.9840
Anti-platelet use, n (%)	395 (46.8)	300 (44.9)	95 (53.7)	0.038

Comparison of baseline demographic and imaging characteristics between those with and without cognitive impairment prior to their qualifying event. Percentage values were calculated using the total number of patients for whom data was available as the denominator. p values are from independent t-tests (age, educational age), Mann-Whitney U test (NIHSS), Fisher's exact test (previous intracerebral haemorrhage) or chi-squared tests (remainder). AF, atrial fibrillation; IQR, interquartile range; NIHSS, National Institutes of Health Stroke Scale; SD, standard deviation.

Table S2. Comparison of imaging features between those and without pre-existing cognitive impairment.

		All	Pre-existing cognitive impairment		p value
			Absent	Present	
n (%)		872	689 (79.0)	183 (21.0)	-
Imaging evidence of previous cortical infarct, n (%)		130 (14.9)	93 (13.5)	37 (20.3)	0.021
Lacunes, presence, n (%)		132 (15.4)	97 (4.2)	35 (19.7)	0.073
pvWMH grade, n (%)	0	531 (60.9)	446 (64.7)	85 (46.5)	<0.00001
	1	166 (19.0)	124 (18.0)	42 (23.0)	
	2	141 (16.2)	97 (14.1)	44 (24.0)	
	3	34 (3.9)	22 (3.2)	12 (6.6)	
dWMH grade, n (%)	0	396 (45.4)	337 (48.9)	59 (32.2)	<0.00001
	1	337 (38.7)	253 (36.7)	84 (45.9)	
	2	95 (10.9)	72 (10.5)	23 (12.6)	
	3	44 (5.1)	27 (3.9)	17 (9.3)	
CSO-PVS grade, n (%)	0	50 (5.9)	38 (5.6)	12 (6.9)	0.9310
	1	375 (44.0)	298 (44.0)	77 (44.0)	
	2	261 (30.6)	212 (31.3)	49 (28.0)	
	3	142 (16.7)	111 (16.4)	31 (17.7)	
	4	24 (2.8)	18 (2.7)	6 (3.4)	
BG-PVS grade, n (%)	0	61 (7.1)	47 (6.9)	14 (7.8)	0.0422
	1	624 (72.1)	508 (74.2)	116 (64.4)	
	2	141 (16.3)	104 (15.2)	37 (20.6)	
	3	36 (4.2)	23 (3.4)	13 (7.2)	
	4	3 (0.4)	3 (0.4)	0 (0.0)	
MTA grade, n (%)	0	193 (24.3)	169 (26.6)	24 (15.1)	<0.00001
	1	375 (47.2)	311 (49.0)	64 (40.3)	
	2	162 (20.4)	120 (18.9)	42 (26.4)	
	3	50 (6.3)	31 (4.9)	19 (12.0)	
	4	14 (1.8)	4 (0.6)	10 (6.3)	
GCA grade, n (%)	0	285 (33.1)	236 (34.7)	49 (27.2)	0.106
	1	378 (43.9)	300 (44.1)	78 (43.3)	
	2	184 (21.4)	137 (20.1)	47 (26.1)	
	3	14 (1.6)	8 (1.2)	6 (3.3)	
cSS, presence, n (%)		1 (0.1)	1 (0.2)	0 (0.0)	1.000
CMB, presence, n (%)		173 (19.8)	133 (19.3)	40 (21.9)	0.441
Presence of >1 CMB, n (%)		77 (8.8)	55 (8.0)	22 (12.0)	0.087

Percentage values were calculated using the total number of patients for whom data was available as the denominator. p values are from Mann-Whitney U tests (pvWMH, dWMH, CSO-PVS, BG-PVS, MTA and GCA grades), Fisher's exact test (cSS) or chi-squared tests (remainder). BG-PVS, MRI-visible perivascular spaces in the basal ganglia; CMB, cerebral microbleed; CSO, MRI-visible perivascular spaces in the centrum semi-ovale; cSS, cortical superficial siderosis; dWMH, deep white matter hyperintensities; GCA, global cortical atrophy; MTA, medial temporal atrophy; pvWVH, periventricular hyperintensities.

Table S3. Multivariable logistic regression for imaging predictors of pre-existing cognitive impairment.

	OR	95% CI	p value
Imaging evidence of previous cortical infarct, presence	1.27	0.79 to 2.02	0.326
Lacunes, presence	1.47	0.94 to 2.31	0.093
pvWMH, per grade increase	1.32	1.08 to 1.61	0.006
dWMH, per grade increase	1.29	1.05 to 1.60	0.016
BG-PVS, per grade increase	1.03	0.77 to 1.36	0.854
MTA, per grade increase	1.55	1.25 to 1.94	<0.0001
GCA, per grade increase	1.09	0.85 to 1.39	0.503
CMB, presence	0.90	0.57 to 1.40	0.629
Presence of >1 CMB	1.13	0.63 to 2.05	0.679

Each model considered only a single neuroimaging marker at a time. All remaining models were adjusted for age, sex, hypertension, diabetes mellitus, heart failure, known AF, educational age, and anti-platelet use. BG-PVS, MRI-visible perivascular spaces in the basal ganglia; CI, confidence intervial; CMB, cerebral microbleed; dWMH, deep white matter hyperintensities; GCA, global cortical atrophy; MTA, medial temporal atrophy; OR, odds ratio; pvWVH, periventricular hyperintensities.

Table S4. Logistic regression models reviewing associations between IQCODE-defined pre-existing cognitive impairment and functional outcome at 24 months.

	Univariable OR (95% CI)	p value	Adjusted OR (95% CI)	p value
Functional dependence (mRS > 2)	2.78 (1.88 to 4.10)	<0.0001	3.33 (1.72 to 6.42)	<0.0001

Multivariable model adjusted for age at event, sex, hypertension, hypercholesterolaemia, diabetes mellitus, smoking, heart failure, clinical history of previous ischaemic events, educational age, admission NIHSS, anti-platelet use, pre-event mRS and the presence of an acute DWI lesion at study entry. CI, confidence interval; DWI, diffusion weighted imaging; MoCA, Montreal Cognitive Assessment; mRS, modified Rankin scale; NIHSS, National Institutes of Health Stroke Scale.
